# Patient and carer perceptions of video, telephone and in-person clinics for Phenylketonuria (PKU)

**DOI:** 10.1186/s13023-024-03295-7

**Published:** 2024-08-20

**Authors:** Hannah McBride, Sharon Evans, Alex Pinto, Anne Daly, Catherine Ashmore, Fatma Ilgaz, Suzanne Ford, Sharon Buckley, Anita MacDonald

**Affiliations:** 1 Birmingham City University, Birmingham, UK; 2 Birmingham Women’s and Children’s NHS Foundation Trust, Birmingham, UK; 3 Department of Nutrition and Dietetics, Faculty of Health Sciences, Hacettepe University, Ankara, 06100 Turkey; 4 NSPKU National Society for Phenylketonuria, London, UK; 5 North Bristol NHS Trust, Bristol, UK; 6 Department of Psychology, Faculty of Health, Psychology and Social Care, Manchester Metropolitan University, 53 Bonsall Street, Manchester, M15 6GX UK

**Keywords:** Phenylketonuria, PKU, Review clinic, Video clinics, Telephone clinics, In-person clinics

## Abstract

**Background:**

In phenylketonuria (PKU), attending multidisciplinary clinic reviews is an important aspect of life-long care. Since the COVID-19 pandemic, video and telephone clinics are used as alternative methods for people with PKU to have contact with their care team. There is limited research concerning patient preference, experience and perceptions of alternative types of clinic review. Individuals from the UK with PKU and their caregivers were invited to complete an online questionnaire, hosted on the National Society for PKU (NSPKU) website and social media platform.

**Results:**

Data was available from 203 respondents. Forty one per cent of respondents (*n* = 49/119) preferred in-person clinics; 41% (*n* = 49) a hybrid of in-person, video and telephone clinics; 9% (*n* = 11) video clinics only, 6% (*n* = 7) telephone only and 3% (*n =* 3) were unsure. The main respondent obstacles to in-person clinics were costs, travel and time, but this was balanced by the benefits of a physical examination and better patient engagement/motivation. Twenty one per cent (*n* = 36/169) of respondents were uncomfortable with the number of healthcare professionals (HCPs) in a clinic room. Patients were less likely to consult with a doctor on video (64%, *n* = 91/143) or phone (50%, *n =* 59/119) reviews compared to in-person (80%, *n* = 146/183). Issues with video and telephone reviews included the shorter time length of review, distractions, technical issues and poor patient engagement.

**Conclusions:**

Online video and telephone clinic platforms were effective in overcoming the challenging circumstances in management, monitoring and treatment of patients with PKU during the COVID-19 pandemic. However, in-person clinics remain the preferred respondent option. It is important that HCPs are flexible, enabling people with PKU a choice of clinic options according to their individual clinical need and circumstances.

**Supplementary Information:**

The online version contains supplementary material available at 10.1186/s13023-024-03295-7.

## Background

Phenylketonuria (PKU) is a rare, metabolic disorder, caused by a deficiency of the enzyme phenylalanine hydroxylase (PAH) that catalyses the hydroxylation of phenylalanine (Phe) to tyrosine. The non-functioning PAH enzyme causes accumulation of Phe in the blood and body tissues and if left untreated, it compromises early development, and results in severe injury to the central nervous system, manifested by severe neurological and cognitive impairment. The management aim is to lower and maintain blood Phe concentrations within a strict target therapeutic range and treatment should commence within the first two weeks of life [1].

The outcome of PKU has improved with early and lifelong management. The primary treatment for PKU is a stringent Phe restricted diet supplemented with a low Phe/Phe free protein substitute and special low protein foods. Pharmaceutical treatments may be used in combination with a Phe restricted diet. These include: sapropterin dihydrochloride and pegvaliase (Palynziq^®^). Sapropterin is a synthetic form of tetrahydrobiopterin (BH4), a natural cofactor for the enzyme PAH. This drug can activate residual PAH enzyme, but as it relies upon the presence of functional PAH, it is only effective in around 30% of patients. Pegvaliase is derived from phenylalanine ammonia lyase, an enzyme that catalyzes the degradation of Phe to ammonia and trans-cinnamic acid. It is licensed for patients aged ≥ 16 years with inadequate blood Phe control (> 600 µmol/L) [2] but it is not available in the UK.

Despite the success of newborn screening, individuals with early-treated PKU do remain at risk of lower intellectual functioning together with impairments in executive function, processing speed, sustained attention, fine motor control, learning capacity, and educational performance, particularly if under-treated [3–9]. Early and continuously treated individuals may not attain their full neurodevelopmental potential and studies often report increased social, behavioural, and/or emotional problems compared with the general population [9–16]. Dietary Phe restriction may be associated with nutritional imbalance and dietary adherence is challenging. Use of adjunct drug therapy is not without risk and requires careful supervision. Patients may also be at increased risk of co-morbidities such as obesity, metabolic syndrome and disordered eating [17–22]. During pregnancy, sub-optimal blood Phe control may increase the risk of maternal PKU syndrome, leading to microcephaly, intellectual disability, growth retardation and congenital heart defects in the infant [23–31]. Therefore, regular and rigorous follow-up is recommended as patients remain vulnerable to mental health disorders, deficits in neurocognitive functioning [1] and maternal PKU syndrome.

Part of the ongoing care and monitoring of PKU involves regular attendance at outpatient clinics, where patients are commonly reviewed by a specialist PKU team of clinicians, dietitians and nurses. Clinic visits may be supplemented with home visits by a dietitian or nursing team and patient in-person events such as family conferences, cooking /educational schools or social functions. Blood Phe levels (from blood spots taken at home and returned to the hospital) are regularly monitored. Target blood Phe levels between 120 and 360 µmol/L for patients ≤ 12 years, pregnancy and pre-conception and between 120 and 600 µmol/L for patients older than 12 years are indicators of good metabolic control [1]. Nutritional monitoring is important to ensure satisfactory growth and development and there are no nutritional imbalances [32]. Adherence to dietary treatment commonly decreases during adolescence and extra observation may be necessary [33].

In 2020, with the onset of the COVID-19 pandemic [34], clinic reviews for PKU were rapidly transferred to remote video/telephone (virtual) clinics to enable continuity of care [35]. This allowed healthcare professionals (HCPs) to make assessments, collect some of the required monitoring data, and educate caregivers and patients [36]. Recent evidence has shown virtual clinic care to be safe and effective, providing a convenient and efficient service at a low cost, reducing journeys by road and travel costs [37]. Virtual care results in fewer missed days off work or school, patients are consulted in their own homes, and they may perceive their HCPs to be more accessible. It has even been associated with improved blood Phe control in PKU [38], by enhancing motivation associated with increased access to care [39]. However, the quality of services provided virtually is variable and can impact the effectiveness of condition management and subsequently reduce patient satisfaction [40]. Some hospitals have invested inadequately in additional patient communication technologies, patients may have unsatisfactory access to suitable equipment and factors such as poor internet connectivity are a common hindrance to successful virtual care [36, 41–44]. A systematic review by Yi et al. [45] highlights the barriers of using telemedicine in older adults and patients with cognitive impairment. In PKU, patients require neurological examination, venous blood samples and anthropometric assessments some of which can only be done during in-person clinics [46, 47] or utilising local laboratories [36]. Some health professionals report difficulties in assessing patient/caregiver body language or building patient rapport with online consultations [36]. Some countries may lack a telemedicine infrastructure [39].

In the UK, prior to the COVID-19 pandemic, outpatient services in PKU had changed little over time. Outpatient non-attendance rates may be higher in the socially disadvantaged, those with poor blood Phe control, and patients who travel long distances to attend specialist PKU clinics. The UK’s National Information Board suggested that a different kind of health service was needed, instructing that the traditional outpatient consultation will become increasingly obsolete [48]. Generally, prior to the COVID-19 pandemic, there was a policy drive by the UK National Health Service (NHS) to harness the potential of digital technologies to improve care models and redesign care pathways in a way that improved the accessibility and efficiency of services and maximize the potential for patient self-management [49]. It was always understood that just installing computers without training the workforce would not allow the system and its people to achieve the new technologies optimal potential [50]. Following the pandemic, the NHS were determined to ensure that ‘virtual’ care remained a routine part of care provision [51].

Therefore, in PKU, it is not surprising that since the pandemic, video and telephone clinics have remained part of routine care. Despite this trend, there is a paucity of scientific studies appraising the use of virtual clinics in PKU. It is therefore important to understand the patient/caregiver experience and how they view the quality of care when delivered by video clinics and /or telephone calls. This study was designed to assess the current perspective of patients with PKU and their caregivers about video/telephone clinics compared with conventional in-person clinics.

## Materials and methods

An online survey (*Jisc Online Surveys*, 4 Portwall Lane, Bristol BS1 6NB, UK; https://www.onlinesurveys.ac.uk*)* accessed via the UK National Society for PKU (NSPKU) website was promoted on social media sites for PKU in order to collect cross-sectional data. The unvalidated questionnaire was designed by a master’s degree student (HM) and edited by experienced inherited metabolic disease (IMD) clinical and research dietitians (AP and AM). Online questionnaires were used in order to reach a wider PKU community across the UK [52]. The questionnaire was pilot tested in the user group prior to study commencement to check for clarity and was then available for completion online from May to September 2021.

### Questionnaire

The online questionnaire addressed patient and caregiver experience and perceptions of in-person, video and telephone review clinics. It comprised 7 demographic questions, 35 multiple choice (17 with extended response) and 6 open ended questions (see supplementary data for questionnaire).

### Data analysis

Qualitative data was analysed thematically to summarise key features of the data collected and highlight similarities and differences between participant perspectives and generate unanticipated responses [53]. The main themes examined and compared across clinic types included: interaction with doctors and other healthcare professionals, length of clinics, the nature of clinic reviews (e.g., discussion of sensitive issues, levels of stress and usefulness) and technical problems with virtual clinics (video and telephone). Quantitative data was analysed by calculating percentages of each response and then Chi Square tests were used to determine if there were significant differences (95% CI, *p* < 0.05) between the responses for the 3 clinic types for certain questions using GraphPad Prism version 10.2.2 for Windows, GraphPad Software, Boston, Massachusetts USA, www.graphpad.com.

### Ethical approval

#### Ethical approval

This was obtained from Birmingham City University (McBride/#9311/sub2/R(A)/2021/Apr/HELS FAEC). Each participant was provided with an online participant information sheet and consent form and asked to confirm consent to participate by selecting a box on the questionnaire.

## Results

### Demographic data

There were 203 UK respondents to this online questionniare: 69% (*n* = 141) were parents/carers of a child with PKU under the age of 18 years; 23% (*n* = 47) adults with PKU (≥ 18 years); 5% (*n* = 10) parents/carers of an adult with PKU (≥ 18 years); and 2% (*n* = 5) adolescents with PKU (aged 12–18 years). Of the individuals with PKU represented in the survey (those responding and those for whom a parent/carer was responding) 53% (*n* = 107) were female and 46% (*n* = 93) were male; 49% (*n* = 100) were < 12 years of age, 27% (*n* = 54) were 12–16 years, 9% (*n* = 18) 19–30 years, 14% (*n* = 29) 31–50 years, and 1% (*n* = 2) 51–60 years of age.

Three quarters (75%, *n* = 152) of respondents accessed paediatric PKU services, and one quarter (25%, *n* = 51) adult services. 15% of respondents (*n* = 31) did not speak English as their first language, with 6% (*n* = 12) responding that they required a translator for clinic appointments.

### “In-person” clinic reviews

Prior to the COVID-19 pandemic (March 2020), 90% (*n* = 183) of respondents had attended in-person clinic reviews with a PKU metabolic team. Those who had not were generally born post March 2020.

Measurement of weight and height and discussions about symptoms and concerns were the main tasks and focus at in-person clinic reviews (Table 1). 68% (*n* = 121/179) of respondents who had attended in-person clinics also reported being given samples of low protein foods and protein substitutes at clinic reviews either by dietitians or dietary product companies.

**Table 1:** Activities conducted during an “in-person” clinic review

Task	% (*n* = 183)
Weight	98 (179)
Height	91 (167)
Discussion of symptoms/concerns	83 (152)
Receive samples of low protein foods or protein substitute	68 (121)
Blood samples taken	36 (65)
Blood pressure checked	16 (30)
Physical examination	14 (26)
Psychometric testing with psychologist	1 (2)
Other – discuss diet/food, taste new low protein products, discuss schools or prescriptions	4 (8)

Over one third (36%, *n* = 65/183) of respondents reported that attending in-person clinic reviews was difficult or very difficult due to geographical access, transport difficulties or distance to travel: *‘We live a long way from the hospital*,* so it is nearly a day’s outing to come’.* 36% (*n* = 66/183) found travel expenses and/or parking charges too expensive with comments such as: *‘It costs a lot of money for petrol and parking costs.’* Having to take time off work and school was a concern for some: *‘Have to get half a day off from work unpaid to get a 2 hour train to the hospital and 2 hour train back*,* for a 15 minute appointment’.* Arranging childcare or travelling with children was considered difficult: *‘It is not too far away but difficult to organise 4 children and find child care’.* Others were less concerned about the travel costs and time taken to travel to in-person clinics: *‘It takes us 4 hours to drive to a clinic*,* but we would rather do this and be seen properly than have a review online like a business meeting’.*

Adults found in-person clinics motivating with comments such as ‘*I do find them extremely useful particularly when I am struggling with following diet. After a visit and discussing with the dietitian and doctor I get a boost and eagerness to start being stricter with following the diet.*’

### Patient experience of doctors in review clinics

80% of respondents (*n* = 146) said that they always saw and spoke to a doctor at each in-person clinic review, but only 64% (*n* = 91) for video reviews and 50% (*n* = 59) for phone reviews (*p* < 0.0001) (Table 2). However, people did comment that: ‘*the consultant is only there if you have had issues with blood levels’* but *‘if I was concerned*,* I could request an appointment’.* There were also concerns that the doctor was different at every clinic irrespective of the clinic type: ‘*We always see a medic and dietitian. The dietitian is the same*,* but the medic may be different’.* Respondents commented that they did not always consider that their doctors viewed their health concerns seriously, ‘*I always feel brushed off by the doctor when I mention any of my health concerns regarding ageing with PKU’* or they felt hurt when unwarranted statements about adherence to treatment was made in clinics: ‘*the registrar asked me if I eat chicken and if I cheat.*”

**Table 2:** Nature and timing of different clinic reviews by type (in-person, video, telephone)

	PKU clinic review type			
	In-person % (*n* = 180–184)*	Online Video % (*n* = 140–143)*	Telephone % (*n* = 113–119)*	*p* value**
**Attendance at clinic reviews**	98 (181/184)	70 (143/203)	59 (119/203)	N/A
**Always see/speak to a doctor**	80 (146)	64 (91)	50 (59)	< 0.0001
**No. HCPs in the room at clinic review in addition to doctor**				
None One Two Three Four ≥Five Varies (1–4) Not reviewed by a medical doctor	- 15 (28) 43 (79) 19 (35) 17 (31) 2 (3) 3 (5) -	2 (3) 42 (60) 34 (48) 12 (17) 4 (6) 3 (4) 0 (0) 3 (5)	2 (2) 66 (75) 23 (26) 5 (6) - - - 3 (3)	< 0.0001
**HCPs present in the same room at the clinic review**				
Doctor Dietitian Nurse Medical trainee Psychologist Biochemist Unsure who they are Other e.g. midwife, advanced practitioner, PKU coordinator, dentist, pharmacist, adult PKU team, phlebotomist	93 (168) 56 (101) 49 (89) 31 (56) 1 (1) - 1 (1) 4 (7)	64 (89) 81 (113) 25 (35) 8 (11) 1 (1) - 1 (2) 1 (2)	57 (66) 63 (72) 17 (19) 3 (3) - 1 (1) 3 (3) 1 (1)	< 0.0001
**Which HCPs do you see at the clinic review (but not necessarily in the same room at the same time)**				
Doctor Dietitian Nurse Medical trainee Psychologist Biochemist Not sure who they are Other (midwife, phlebotomist, advanced practitioner, clinical support worker)	91 (166) 97 (177) 40 (74) 31 (56) 2 (3) 1 (1) - 3 (6)	N/A	N/A	N/A
**Total time spent in clinic review**				
≤ 15 min ≤ 30 min 30 min to 1 h 1 to 2 h > 2 h	10 (18) 24 (44) 34 (63) 29 (53) 4 (7)	22 (31) 41 (58) 34 (49) 3 (4) -	49 (57) 33 (38) 16 (19) 2 (2) -	< 0.0001
**Opinion on the length of the clinic review**				
Just right Too short Too long Don’t know	83 (151) 8 (15) 5 (10) 4 (7)	70 (100) 20 (28) 3 (5) 7 (10)	68 (79) 23 (27) 2 (2) 7 (8)	0.03
**How stressful/relaxed are clinic reviews**				
Stressful Slightly stressful Neutral Relaxed Very relaxed	12 (21) 26 (47) 26 (48) 23 (41) 14 (25)	8 (12) 28 (40) 27 (38) 27 (38) 10 (14)	6 (7) 19 (22) 37 (43) 27 (31) 12 (14)	0.48
**How useful are clinic reviews**				
Very useful Useful Neutral Not useful Don’t know	50 (92) 38 (69) 8 (14) 4 (8) -	30 (43) 36 (52) 19 (27) 13 (19) 1 (2)	29 (34) 27 (31) 31 (36) 14 (16) -	< 0.0001
**Sensitive topics are discussed at clinic reviews**	24 (44)	21 (29)	12 (14)	0.08

*Note the number of respondents varied by question; *PKU* Phenylketonuria, *HCP* Healthcare Professional

**Chi-square test; *N/A* not applicable

### Patient experience of other healthcare professionals in review clinics

Patients were statistically (*p* < 0.0001) more likely to see other HCPs as well as the doctor if they attended in-person clinics rather than video or telephone clinics. 81% of respondents (*n* = 148) said that they saw at least two other HCPs in addition to a doctor at their in-person clinic visits (mostly dietitians and nurses), compared to 53%, *n* = 75 for video calls, and 28%, *n* = 32 for telephone calls (Table 2). When asked how they felt about the number of HCPs in the room, 21% (*n* = 36/169) said they felt uncomfortable, stressed or overwhelmed. This was mainly for in-person reviews, as video and telephone reviews rarely had more than 2 health professionals in attendance. Verbatim comments included: ‘*In a room with a lot of people it feels judgmental*,*’ ‘We attempt to* [discuss sensitive issues] *but I think the number of people in the room stops my daughter talking about the things that she wants/needs to*,*’ ‘Often the different people are not introduced and people think it is okay to come in and out*,*’ ‘there are too many people. It is not good for my teenage boy having to speak up for himself.’* However, some people found it beneficial to speak to a group of HCPs with comments such as ‘*it saves time as I don’t have to repeat the same information to others’.*

For in-person reviews, participants reported there was one main clinic room and doctors were the most likely HCP to be present (93%, *n* = 168) followed by dietitians (56%, *n* = 101) and nurses (49%, *n* = 89), but in video/telephone clinics, the dietitian was the most likely to be present (81%/63%), followed by doctors (64%/57%) (*p* < 0.0001) (Table 2). This was apparent in the verbatim comments: ‘*Always speak to the medic in in-person clinic but video clinics are dietetic/nurse led so do not speak to medics then’.* For in-person clinics, dietitians were the HCP most likely to be seen (97%, *n* = 177), followed by doctors (91%, *n* = 166) and nurses (40%, *n* = 74), but they sometimes saw patients individually in a different room rather than in a setting together with other professionals. Comments included: ‘*We have several appointments …. some are with doctors present*,* some are with just dietitians*,* both are equally important.’*

### Length of clinic reviews

The average length of time spent on clinic reviews was significantly higher (p < 0.0001) for in-person clinics with one third taking 30–60 min and the remaining spread mostly between ≤ 30 min and 1–2 h (Table 2). Video reviews were more likely to be shorter with 41% (*n* = 58) taking 15–30 min and one third (*n* = 49) 30–60 min. Telephone reviews were also shorter, with half (*n* = 57) taking less than 15 min and one third (*n* = 38) 15–30 min. Most respondents thought these times were adequate for each of the clinic types, although they were more likely to be happy with the length of in-person clinics (p = 0.03) than video or telephone clinics and 20% (*n* = 28)/23% (*n* = 27) felt that video/telephone reviews were too short. Verbatim comments about in-person clinics included: *‘The wait time is too long but the review itself is just right’* for video reviews *‘‘they always feel shorter and not as relaxed as in-person’ ;’* and telephone reviews *‘it’s difficult to have a long conversation over the telephone when you cannot see their face and react to their body language.’*

### Nature of clinic reviews

Patients were statistically (*p* < 0.0001) more likely to find in-person clinics useful or very useful (88%, *n* = 161) compared with video (66%, *n* = 95) or telephone reviews (56%, *n* = 65) (Table 2). There was no difference between review type for any stress experienced (*p* = 0.48). For in-person reviews, the main stress centred around travel, parking, taking time off work/school, childcare and discussions concerning blood Phe control. For video and telephone reviews, issues with technology and unwelcome distractions caused stress.

Sensitive topics such as drugs, alcohol and sexual health were less likely to be discussed during a telephone call (12%, *n* = 14) compared with in-person (24%, *n* = 44) or video reviews (21%, *n* = 29), although this difference was not statistically significant (*p* = 0.08) (Table 2).

### Technical issues associated with video and telephone clinic reviews

When asked about technical issues experienced with video and telephone clinic reviews, 57% (*n* = 70/122) reported difficulties with video, and 49% (*n* = 56/115) with telephone reviews. This included difficulties with establishing a connection, issues with the device, issues with the internet and inability to see and/or hear the HCPs (Table 3).

**Table 3:** Technical problems with video or telephone clinic reviews

Technical issue	Online video % (*n* = 122–137)*
Cannot see all the health professionals involved in the consultation	27 (37)
Cannot hear the health professionals involved in the consultation	27 (36)
Technical issues with the internet	26 (35)
Difficulty establishing an online connection	21 (28)
Technical issues with online device used e.g. computer	20 (27)
Difficulty with technology at home to allow a videocall e.g. internet, laptop or tablet	14 (18)
Difficulty accessing a phone connection	13 (18)
Other (e.g. cable pulled out by accident)	4 (6)
No difficulties	43 (52)
**Verbatim comments:**	
• *Link for video call does not always come through.*	
• *I was on my phone and the battery ran out*,* the consultation was very disjointed.*	
• *Can be difficult with interpreter.*	
• *There’s too much delay in people talking and the screen also freezes so you don’t have a free flowing conversation.*	
**Technical issue**	**Telephone** **% (*n*** = 103–115)*
Cannot hear all the health professionals involved in the consultation	17 (18)
Difficulty with technology at home to allow a telephone call (e.g. no landline, no mobile phone, phone not working)	10 (10)
Difficulty accessing a phone connection	9 (10)
Other: e.g. HCP rang wrong phone, no phone credit	12 (14)
No difficulties	51 (59)
**Verbatim comments:**	
• *Run out of credit.*	
• *Not knowing exactly when the call may come from the hospital is a problem.*	

*Note the number of respondents varied by question

When asked what type of clinic they would prefer to attend, 41% (*n* = 49/119) of respondents said in-person and a further 41% (*n* = 49) said a hybrid of in-person, video and telephone. 9% (*n* = 11) preferred video only, 6% (*n* = 7) telephone only, 1% a hybrid of video and telephone (*n* = 1), and 2% (*n* = 2) were unsure. Table 4 shows some of the comments from respondents.

**Table 4:** Other general pros and cons of clinic types – verbatim comments from users

Verbatim comments regarding “in-person” review clinics	
**Pros**	**Cons**
I find it allows the health professionals to see how my little boy is developing. In-person is essential as my daughter does not know anyone else who has PKU, therefore, this is her only recognition that she is not alone. I think the team build better relationships with child patients by seeing them in person. In-person clinics motivate my daughter more even though video clinics are easy. These are better for children as it’s not easy to get a child to sit still for a video call. We are able to discuss issues, often company reps are in clinic too, so we get the samples of new products. Body language helps people to settle. I think there is a need for in-person when discussing important and sensitive information.	Most of the stress comes with taking time off work, juggling child care, taking time off schools. Schools do not like children taking time off. Actual appointment good. But not worth whole day with delays in appointment and travel. I’m not sure it is needed for every appointment. Maybe alternate between visiting and virtual. They are not useful when a child is well controlled and neither parents nor MDT have concerns - why take a child out of school for a morning to say “it all seems fine". We do not get as much out of clinics as when she was younger.
**Verbatim comments regarding VIDEO review clinics**	
**Pros**	**Cons**
Being able to have the clinic when I haven’t been worn out with travel and when I’m in a physically comfortable space means I’m much more able to concentrate, and therefore express myself better and take in what is said more accurately. I think a mix of video and in-person would be a good balance. It is good to have the flexibility a video call provides, especially with both parents working full time. Less under pressure. Children’s behaviour better. Can think better and ask questions. The video calls are more convenient when there are no issues. It’s a better option that doesn’t affect his school day as much. Perfectly fine when an in-person meeting is not an option.	I can’t hear or see people properly and I feel I don’t get time to say what I need to say. Would be a poor long term solution. In person is a must. I don’t have my usual bloods and health physical health checks like I would have done if the clinic was in-person. My daughter does not engage on video calls. It’s stressful trying to deal with my other child and the phone ringing or the door going. I find that they are very impersonal and the health professionals only talk to me and not directly to my child. Worry in case we lose connection or about getting onto the video call. Doesn’t compare to the in-person activities at group clinic.
**Verbatim comments regarding TELEPHONE review clinics**	
**Pros**	**Cons**
Easier conversation and just the right time without any waiting. Always more relaxed. It’s easier as I don’t have to go out of my way to get there. My kids can also carry on with their normal stuff of playing instead of sitting in a room getting bored.	Waste of time as the doctor spoke to me and not my teenager. In-person is preferable, as a lot of information is missed on the phone - appointment feels rushed. Forget half of what I need to say. My husband keeps information to himself.

## Discussion

For patients with PKU, clinic review is an intrinsic part of continuing care by their health professional providers. This study explored the views and experiences of individuals with PKU and their parents/carers when attending either in-person, video or telephone clinics. Although there is increasing pressure to embrace video and telephone clinics because of their ease of use, low cost, and the increased access to care, patient satisfaction with the type of service provision should remain a priority. Video and telephone clinics must provide the same, or ideally an improved level of care, to be considered a suitable option by service users. In this survey, most respondents expressed a desire for in-person clinics to continue, but considered the adoption of a hybrid approach with either video or telephone clinics was appropriate. As well as ensuring that clinical care standards are maintained, the type of clinic attended ought to be influenced by individual circumstances and experiences, indicating a need for flexibility regarding the type of clinic review.

Most respondents reported attending in-person clinics at the hospital with a PKU metabolic team prior to the COVID-19 pandemic, so they had knowledge of the benefits and drawbacks of this model of care. The respondents allocated a higher “usefulness” rating to in-person clinics; they considered that in-depth patient issues were better managed. For in-person clinics, patients were more likely to be reviewed by a medical doctor (80%, compared with 64% in video clinics and 50% by telephone review). In particular, caregivers considered that in their child’s clinic review, it was essential that they received a clinical examination, observation and monitoring. Assessing child development is more challenging by video or telephone review and caregivers found in-person clinics better for discussing any developmental concerns. They also felt that in-person clinics were central to their child’s PKU education and motivation. It was considered important that children received information about PKU and its treatment directly from the HCPs, as they listened more intently and regarded their condition more seriously. Some PKU teams also ran separate education and cooking sessions for the children within the clinic. They also had the opportunity to meet other children with PKU at the hospital. Adults with PKU found direct contact with their HCPs motivational and it helped them maintain or improve their treatment plan. Caregivers and patients reported that they found it easier to talk to HCPs in-person compared with video and telephone clinics. They said they were able to consider more aspects of PKU care. Respondents described the importance of the rapport and relationship they had built with HCPs over many years. When this had been established, their in-person clinic consultation experience felt comfortable, more amiable and they expressed more confidence in their HCPs. They were less satisfied when they saw different or junior medical doctors in clinics or if the HCPs demonstrated little understanding or empathy about the issues they faced. A minority of respondents commented that clinics were repetitive, and they gained little by attending in-person clinics. They felt that their condition was stable and there was rarely any change to their management plan.

In the in-person clinics, 41% of respondents described that there were commonly three or more people present in the doctors’ consultation room including a nurse, trainee doctors as well as medical consultants, but in video or telephone clinics patients were more likely to be reviewed by the medical doctor and dietitian only. Although most respondents tolerated high numbers of professionals in the same room, 21% were overwhelmed, felt awkward and self-conscious, particularly if some HCPs just observed the review, with no apparent contribution. Some said that it felt judgmental when discussing blood Phe control in a room full of people. Others reported that it was not helpful to have a room full of HCPs in transition clinics for teenagers, when they were expected to speak up for themselves without caregivers present. Sensitive issues were discussed in less than 30% of all clinics (in-person, video and telephone) and topics such as women’s reproductive health and social issues such as drugs and alcohol were even less likely to be covered by telephone clinics. Although it is important for trainee HCPs to observe clinic interactions, there should be a limit defining the number of professionals who are present in one clinic room, with the priority on ensuring that patients/caregivers are comfortable and feel able to talk about sensitive and difficult matters. All patients/caregivers should be given the opportunity to consent to observers attending their clinic review.

Many respondents reported that they saw their dietitian in a separate room from the medical doctor for in-person clinics. They found this acceptable, as they were usually given more time to review dietary management and ask questions, as necessary. They appreciated that the dietitian was able to explain or reinforce information given by the medical doctor. They particularly valued information on the suitability of new dietary products and protein substitutes although some parents commented that they had gained the same information earlier via social media. They welcomed receiving samples of special low protein foods and protein substitutes within clinics. Some patients said they prepared thoroughly before in-person clinics, writing down any questions or concerns.

Although many respondents said their in-person consultations were relaxed and informal, some respondents had anxiety about their clinic appointments. They worried about the travel to the hospital, any negative opinion that may be expressed by the health professionals, and the behaviour of their child in clinic, particularly when children were reluctant attendees. Some caregivers reported their children were fearful about clinic visits, particularly about venous blood tests. Equally, respondents expressed anxieties associated with video consultations. Some respondents said they suffered with social anxiety, and they found any kind of appointment stressful. Some did not relax because of home distractions e.g., demands of other children, doorbells or telephones ringing. Others felt embarrassed about their children’s lack of co-operation and engagement with video appointments and their failure to be seen or communicate on camera.

Some respondents stated that confidentiality was a problem with video/telephone reviews at home, as they had difficulty finding space to have a private conversation without others listening. Around 25% of respondents said they could not see or hear all HCPs on the video reviews and 18% said they could not hear the conversation by telephone review. Patients for whom English was not their first language were disadvantaged by telephone clinics. They were unable to see nonverbal cues that may help understanding during in-person consultations. Some caregivers said that only one parent was involved with a telephone review (commonly the parent who spoke English) and information was not always shared with both parents.

Undoubtedly in-person clinic appointments caused considerable time pressures due to travel, with 2 to 4 h round trips being common. This has been reported by others [36, 38]. This was affected by the locations of the specialised clinics, and there is an expectation from service providers that patients are prepared to travel [54]. Consequently, this caused a financial burden because of petrol and parking costs, and expenditure on public transport. With video clinics/telephone calls, the time burden of travelling to the hospital, the need to take time off work, and to organise childcare were eliminated. Management of school time was better as some schools are increasingly reluctant to give children time off for hospital appointments in term time. Some adults used their holiday entitlement to attend in-person clinics. However, some respondents who preferred in-person reviews said that appointments were infrequent, and they did not mind being inconvenienced or financially ‘out of pocket’ as they considered ‘in-person’ consultations essential.

Although video and telephone clinics overall rated highly, almost 50% of the respondents preferred in-person clinics only, and almost 50% a hybrid of video/telephone and in-person clinics. Very few respondents suggested that video/telephone clinics should replace in-person clinics completely. Virtual clinics served a purpose during the COVID-19 pandemic [35, 36] but were considered a temporary measure. However, in an Italian study, caregivers or younger patients with PKU were more likely to utilise video clinics due to greater uncertainties in their management, particularly if recently diagnosed, or if there were concerns about growth or metabolic control [55]. There is also evidence that some adult patients with previously poor in-person clinic attendance were more likely to attend video clinics as it integrated better with work life [55]. In our study, video/telephone consultations appeared to work best when respondents already had a good working relationship with their health professional team and felt comfortable in their presence [56]. Respondents considered it was difficult to have in-depth conversations in video/telephone clinics, and information was missed, leaving some respondents feeling quite vulnerable. They said that telephone calls were information gathering for the benefit of the HCPs, but they had less opportunity to be heard and engaged in their own care. Physical examinations could not occur, and reliable measures of assessing height and weight were difficult. Some patients considered that their physical symptoms may be missed or even misunderstood. Caregivers reported that their children were less likely to engage online and some even said their children had become ‘*removed from their condition*’ when they had no direct contact with professionals. Sometimes video calls were stopped and the review transferred to a telephone call due to technical issues. Some respondents ran out of phone credit part way through a review, and this led to some appointments being rescheduled. Overall, technical problems included lack of sound, poor sound quality, and loss of picture on videos. For some families, poor broadband access may limit their ability to effectively engage with video clinics, for multifactorial reasons including geographical location and socio-economic status [57].

Consultation times were shorter with video/telephone clinics. Almost 70% of in-person clinics lasted 30 min or more compared with only 37% of video calls and 18% of telephone calls. In fact, almost 50% of telephone reviews were no longer than 15 min. Many felt the length of time of in-person clinics was about right. They would usually see more than one professional (medical doctor and dietitian, or medical doctor, dietitian and nurse) and time spent with the different professionals would justify the time and expense in travelling to the clinic. With telephone calls, there was inadequate time for respondents to discuss the management plan or ask questions. Some found the telephone reviews so quick that they would forget what they needed to talk about. Consultations that were rushed gave the impression that health professionals were uncaring.

Although healthcare organizations are encouraged to embrace telehealth (video and audio technology), patient experience is a key quality indicator of healthcare. Whilst spending fewer resources on in-person clinics can be beneficial in terms of heavy demand and capacity [54], it can also pose a risk to patients who may not receive the necessary support and have full opportunity to discuss all their issues [58]. Other studies conducted by the NHS [59] have also found that patients prefer in-person clinics. Many patients consider that in video/telephone clinics, a full assessment of clinical problems or symptoms is not taken, and these clinics do not address their needs [60, 61]. Long term outcomes associated with video/telephone clinics require careful study. Although they have valuable potential, it must be carefully considered how they can be successfully incorporated into the patient care pathway for PKU.

Overall, patient/parent experience in PKU clinics can be improved, using a hybrid approach of both in-person and video clinics. To ensure that patients gain maximum value from long and expensive journeys to clinics, frequency of in-person clinics could be reduced but the quality of the review could be enhanced by including comprehensive clinical assessments, psycho-social support, monitoring of well-being, allocated time for patient education and even include elements that encourage patient social interaction. Patients and parents need to develop confidence in their HCPs, and care by a consistent and experienced healthcare team over time is a key component. Transition clinics need consideration in order to find a way of engaging teenagers so that they feel comfortable and in control during a review and not overwhelmed or intimated by the number of HCPs present. They need to be listened to, trusted and taken seriously and facilitated to feel more connected with their condition.

All virtual clinics should be compliant with national and organisational data protection and telehealth regulations [62]. It is important that video/telephone clinics are structured with a clear plan of their aims and expected outcomes. Patients/caregivers should be given plenty of opportunity to outline their health status, progress, concerns, and treatment needs as part of their ongoing management plan. To facilitate effective communication, translation services should be used when necessary. Privacy of the patient should be maintained with other people unable to hear or see the interview. If there are any safeguarding concerns, video consultations should be replaced by in-person clinics [63]. If patients cannot use video technology, then telephone (if appropriate) or in-person clinics should be offered. Video and telephone clinics should not be seen as a ‘tick box exercise’.

### Limitations

Participation in this study was voluntary and respondents were not randomly selected. Additionally, as this was an online survey, individuals without internet access may have been unable to participate. The NSPKU social media platforms were used to promote the survey, meaning participants were more likely to be members of the NSPKU who may be more cognisant and informed about PKU and therefore may not be representative of the entire PKU population. Data collected was based on individual perceptions of the PKU service, and therefore may be subjective. Most respondents to this questionnaire were caregivers of children with PKU. We did not compare paediatric versus adult responses but acknowledge that their needs and concerns differ. In addition, clinic resources vary between clinics, which may have affected the results.

### Implications

Choice of clinic should be determined by its purpose but it is clear that virtual clinics and the use of technology can complement in-person clinics and enable enhanced personalised care. Whilst physical measurements using validated equipment (e.g. height, weight, blood pressure), examinations, blood, urine and other tests should be primarily done via in-person clinics, better use could be made of technology and virtual clinics to enhance patient experience, increase access to HCPs and improve organisation and efficiency of patient care. Virtual clinics provide opportunity for patients to be followed up quickly in response to immediate issues. They reduce geographical barriers and so may reduce missed appointments and help lessen patients lost to follow up. Apps can be used to collect pre-clinic histories, including dietary reports and drug information, with patient/caregiver ability to directly access blood Phe analysis and test results, health reports and treatment plans directly from hospital portal systems. Neuro-cognitive testing or group counselling meetings could be offered online. Delivering online education either via individual or group family teaching sessions is valuable. Utilisation of digital technology for easier access to dietary prescriptions and samples of new low protein foods should enhance patient experience.

However, regardless of clinic type or digital support it is clear that patients/caregivers value direct time with health care professionals discussing their medical and care needs. The length of video/phone clinics should be adequate to meet the individual needs of the patient/caregiver and the environment should enable sensitive issues to be discussed. The development of standard operating procedures for virtual clinics will help to ensure these issues are addressed.

## Conclusions

In summary, this study demonstrates that the patient with PKU and their parent/caregivers’ experience of video and telephone reviews does not surpass that of in-person clinics, despite the time and travel inconvenience involved. They see in-person appointments as an essential component of their individual or child’s clinical management. However, there are advantages to video and telephone reviews and a combination of in-person, video and telephone consultations should be considered. It is likely that offering a choice of different types of clinic review to meet individual needs will lead to improved attendance, adherence and self-efficacy in long term management. Specialist PKU professionals should respond flexibly and adapt to provide broader consultation options to improve the experience for patients with PKU. The challenge is now to combine and embrace the benefits of all clinic review systems so that they become engrained into routine care pathway systems.

### Electronic supplementary material

Below is the link to the electronic supplementary material.

Supplementary Material 1

## Data Availability

The datasets used and/or analysed during the current study are available from the corresponding author on reasonable request.

## Abbreviations

PKU: Phenylketonuria
NSPKU: National Society for Phenylketonuria
HCPs: Health care professionals
PAH: Phenylalanine hydroxylase
Phe: Phenylalanine
NHS: National Health Service
UK: United Kingdom
IMD: Inherited metabolic disorder
BH4: Tetrahydrobiopterin
