# Supplementary material for: Patient and carer perceptions of video, telephone and in-person clinics for Phenylketonuria (PKU)
Source: Orphanet J Rare Dis. 2024 Aug 20;19:303. doi: 10.1186/s13023-024-03295-7 (PMC11337755; doi:10.1186/s13023-024-03295-7)
Supplement: Supplementary file 1 — Supplementary Material 1 [file 13023_2024_3295_MOESM1_ESM.pdf]

Patient and family perceptions of PKU clinics

1. Please tick one of the options below
   - ☐ I am an adult (18 years or over) with PKU
   - ☐ I am a parent/carer of an adult with PKU (18 years and over)
   - ☐ I am a parent/carer of a child with PKU (under 18 years)
   - ☐ I am under 18 years with PKU
   - ☐ None of the above

2. Is the person with PKU a UK resident

   ☐ Yes ☐ No

3. Do you speak English as your first language?

   ☐ Yes ☐ No

4. Do you require a translator during hospital clinic reviews?

   ☐ Yes ☐ No

5. How old is the person with PKU?

   ☐ Less than 12y ☐ 12-18y ☐ 19-30y

   ☐ 31-50y ☐ 51-60y ☐ Over 60y

6. Please select the service you attend

   ☐ Adult services ☐ Paediatric services

7. What is the sex of the person with PKU?

   ☐ Male ☐ Non-binary ☐ Other

   ☐ Female ☐ Prefer not to say

8. Prior to March 2020, did you attend clinic reviews at hospital with a PKU metabolic team?

- ☐ Yes
- ☐ No
- ☐ Other

8.a. If you selected other, please specify:

9. Have you attended any face-to-face clinic reviews for PKU care?

- ☐ Yes
- ☐ No

10. At your clinic visits, do you always see and speak to a doctor?

☐ Yes ☐ No ☐ Sometimes ☐ Don’t know

10.a. Please comment

11. How many health professionals are present in the room when you see the doctor in the clinic?

☐ 1	☐ 3	☐ 5	☐ Other
☐ 2	☐ 4	☐ 6+	

11.a. If you selected Other, please specify:

12. Please select health professionals present in the room (tick all that apply)

☐ Doctor	☐ Dietitian	☐ Biochemist	☐ Other
☐ Nurse	☐ Psychologist	☐ Trainees	

12.a. If you selected Other, please specify:

13. How do you feel about the number of people present in the room?

14. Please select the health professionals you normally talk to at the clinic review

- ☐ Doctor
- ☐ Dietitian
- ☐ Biochemist
- ☐ Nurse
- ☐ Psychologist
- ☐ Other

14.a. If you selected Other, please specify:

15. Please tick which of the following are done during the clinic review (tick all that apply)

- ☐ Height measured
- ☐ Weight measured
- ☐ Blood samples taken to check your nutrition e.g. iron levels
- ☐ Blood pressure check
- ☐ Opportunity to discuss symptoms or concerns
- ☐ Physical examination by doctor
- ☐ Psychometric testing with a psychologist
- ☐ Other

15.a. If you selected Other, please specify:

16. Are sensitive topics discussed during your face-to-face review? (e.g. Drugs, alcohol, sexual health)

- ☐ Yes
- ☐ No
- ☐ Not applicable

16.a. Please comment

17. Are you usually given samples of low protein foods or protein substitute?

- [ ] Yes
- [ ] No
- [ ] Don't know

18. How long on average do face-to-face clinic reviews last?

- [ ] Up to 15 min
- [ ] 30 min – 1 hour
- [ ] Over 2 hours
- [ ] Other
- [ ] Up to 30 min
- [ ] 1-2 hours
- [ ] Don't know

18.a. If you selected Other, please specify:

19. To what extent do you find face-to-face clinic reviews useful? (e.g. able to discuss health needs and problems, ask questions, get practical advice, receive support and reassurance?)

- [ ] Very useful
- [ ] Neutral
- [ ] Don't know
- [ ] Useful
- [ ] Not useful

19.a. Please comment

20. To what extent do you feel stressed or relaxed during the face-to-face clinic reviews?

- [ ] Stressful
- [ ] Neutral
- [ ] Very relaxed
- [ ] Slightly stressful
- [ ] Relaxed
- [ ] Don't know

20.a. Please comment

21. Using the scale below, how easy or difficult is it to travel to the face to face clinics reviews (e.g. transport available, distance to clinic) Please tick.

- [ ] Very difficult
- [ ] Neutral
- [ ] Very easy
- [ ] Difficult
- [ ] Easy
- [ ] Don't know

21.a. Please comment

22. Please select which best describes the travel costs/parking charges associated with face-to-face clinic reviews.

☐ Expensive ☐ Acceptable costs ☐ No costs ☐ Don't know

22.a. Please comment

23. Please give your opinion on the time length of the clinic reviews.

☐ Clinic too long ☐ Clinic just right ☐ Clinic too short ☐ Don't know

23.a. Please comment

24. Please give your overall comments about the face to face review service.

25. Have you attended online video clinics for PKU care?

☐ Yes ☐ No

26. At your video clinic reviews, do you always see and speak to a doctor?

☐ Yes ☐ No ☐ Don't know

26.a. Please comment

27. How many health professionals are in the same room as your doctor during your video clinic reviews? (Please tick)

- ☐ 1
- ☐ 3
- ☐ 5
- ☐ Other
- ☐ 2
- ☐ 4
- ☐ 6+

27.a. If you selected Other, please specify:

27.b. How do you feel about this?

28. Please select health professionals present in the video link during the clinic (tick all that apply)

- ☐ Doctor
- ☐ Dietitian
- ☐ Biochemist
- ☐ Other
- ☐ Nurse
- ☐ Psychologist
- ☐ Trainees

28.a. If you selected Other, please specify:

29. Are sensitive topics discussed during your video clinic review (e.g., Drugs, alcohol, sexual health).

- ☐ Yes
- ☐ No

29.a. Please comment

30. How long, on average, do your video clinic reviews last? (this includes talking to all of the PKU team)

- ☐ Up to 15 min
- ☐ 30 min – 1 hour
- ☐ Over 2 hours
- ☐ Other
- ☐ Up to 30 min
- ☐ 1-2 hours
- ☐ Don’t know

30.a. If you selected Other, please specify:

31. To what extent do you find the online virtual video clinics useful? (ability to discuss health needs, ask questions and get practical advice?)

☐ Very useful ☐ Neutral ☐ Don't know

☐ Useful ☐ Not useful

31.a. Please comment

32. To what extent do you feel stressed or relaxed during the video clinic reviews?

☐ Stressful ☐ Neutral ☐ Very relaxed

☐ Slightly stressful ☐ Relaxed ☐ Don't know

32.a. Please comment

33. Please give your opinion on the length of the video clinic reviews.

☐ Clinic too long ☐ Clinic just right ☐ Clinic too short ☐ Don't know

33.a. Please comment

34. Do you experience any of the following technical problems with video clinic reviews?

	Yes	No	Don't Know	N/A
Difficult to establish online connection				
Difficult to access by phone connection				
Technical issues with online device used e.g., computer				
Technical issues with internet connection				
Cannot see all the health professionals involved in the consultation				
Cannot hear all health professionals involved in the consultation				
Difficulty with technology at home to allow a videocall (e.g., internet, laptop or tablet).				
No difficulties				
Other				

34.a. If other, please specify

35. Please give your overall comments on the video clinic reviews.

36. Have you attended any telephone reviews for PKU care?

☐ Yes ☐ No

36.a. Please comment

37. For your telephone reviews, do you always speak to a doctor?

☐ Yes ☐ No ☐ Don't know

37.a. Please comment

38. How many health professionals are on the same call as the doctor during your telephone review? (Please tick)

☐ 1 ☐ 3 ☐ 5 ☐ Other

☐ 2 ☐ 4 ☐ 6+

38.a. If you selected Other, please specify:

38.b. How do you feel about the number of health professionals on the call?

39. Please select health professionals present on the call (tick all that apply)

☐ Doctor ☐ Dietitian ☐ Biochemist ☐ Other

☐ Nurse ☐ Psychologist ☐ Trainees

39.a. If you selected Other, please specify:

40. Are sensitive topics discussed during your telephone review (e.g., Drugs, alcohol, sexual health).

☐ Yes ☐ No ☐ Not applicable

40.a. Please comment

41. How long, on average, are the telephone clinic reviews? (this includes talking to all of the PKU team)

☐ Up to 15 min ☐ 30 min – 1 hour ☐ Over 2 hours ☐ Other

☐ Up to 30 min ☐ 1-2 hours ☐ Don't know

41.a. If you selected Other, please specify:

42. Do you experience any of the following technical problems with telephone reviews?

	Yes	No	Don't Know	N/A
Difficult to access by phone connection				
Cannot hear all health professionals involved in the consultation				
Difficulty at home to allow a telephone call (e.g., no landline, no mobile, phone not working).				
No difficulties				
Other				

42.a. If other, please specify

43. To what extent do you find the telephone clinics useful? (ability to discuss health needs, ask questions and get practical advice?)

☐ Very useful ☐ Neutral ☐ Don't know

☐ Useful ☐ Not useful

43.a. Please comment

44. To what extent do you feel stressed or relaxed during the video clinic reviews?

- [ ] Stressful
- [ ] Neutral
- [ ] Very relaxed
- [ ] Slightly stressful
- [ ] Relaxed
- [ ] Don’t know

44.a. Please comment

45. Please give your opinion on the length of the video clinic reviews.

- [ ] Clinic too long
- [ ] Clinic just right
- [ ] Clinic too short
- [ ] Don’t know

45.a. Please comment

46. Please give your overall comment on the telephone review service.

47. How would you prefer to attend clinics in the future?

- [ ] Face-to-face only
- [ ] Online virtual video only
- [ ] Online telephone only
- [ ] Mixture of face-to-face, virtual video and telephone
- [ ] Don’t know
- [ ] Other

47.a. If you selected Other, please specify
